# Stroke still strikes hard: we need more primary prevention

**DOI:** 10.1016/j.lana.2026.101537

**Published:** 2026-06-12

**Authors:** The Lancet Regional Health – Americas

Although migraine is the most common neurological disease worldwide, stroke surpasses migraine—and even dementia—in terms of disability burden. Neurological conditions are the leading cause of disability adjusted life years (DALYs); that is, the number of years of healthy life we lose due to illnesses. Stroke accounts for 42% of all neurological DALYs; migraine, 16%; and dementia, 10%. Globally, there are an estimated 12 million new stroke cases each year, and 94 million people currently living have experienced a stroke. Stroke not only has a high impact on disability; it is the third leading cause of death. Encouragingly, most risk factors associated with stroke are preventable.

In our June, 2026, issue, we published a Series on stroke prevention in the Americas. Silva and colleagues report that 1·2 million new cases of stroke and 15·7 million prevalent cases have been reported in the region, based on 2023 Global Burden Disease (GBD) data. These numbers indicate an increase from the 2021 GBD estimates (1·1 million new stroke cases and 12·9 million prevalent cases), an increase of about 9% in new cases and about 22% in prevalent cases. Martins and colleagues focus on the implementation of stroke prevention in the Americas. The authors emphasise that gaps between policy and implementation, along with the lack of allocated resources, are major problems, and multidisciplinary team efforts and shifting tasks are vital to address these issues.

Due to the high burden of stroke, attention is usually focused on care. However, prevention is often relegated, particularly in health-care system planning, even though many stroke risk factors are modifiable, including high blood pressure, high LDL cholesterol, high blood sugar, smoking, high intake of salt, physical inactivity, excess bodyweight, and high alcohol intake. Hypertension, high LDL cholesterol, and high blood sugar are often underdiagnosed. A modest reduction in salt intake leads to blood pressure reduction in individuals with hypertension and even in those with normal blood pressure. Nevertheless, prevention should not be viewed as individuals’ responsibility only, rather needing a wider approach in which primary health-care facilities should play a vital role. These facilities are the first line of attention and provide a key opportunity to identify individuals at potential risk of having stroke and encourage modification of stroke risk factors.

Stroke prevention should not be regarded as mainly the responsibility of neurologists or cardiologists either, although prevention guidelines are typically provided by the American Heart Association and the American Stroke Association. A great example of secondary prevention is the use of barbershops to promote lifestyle modification and doctor appointments among people with hypertension, the strongest modifiable risk factor of stroke. Another example is a community health-worker-led home intervention in Argentina to coach and monitor blood pressure.

Initiatives such as the HEARTS programmes in low-income and middle-income countries and CARDIO4Cities in Brazil, aimed to strengthen primary health-care systems to tackle cardiovascular disease, are showing promising results. Although the HEARTS programme has been implemented in multiple countries, coverage remains limited. For example, HEARTS is currently implemented in 30 countries and more than 10,000 primary health-care facilities in the region, but Brazil alone has 45 700 primary health care facilities (Unidades Básicas de Saúde), underscoring the gap. We have strong guidelines for prevention, but we have still weak implementation. In the Series on stroke prevention, Jürisson and colleagues also comment on the implementation gap for stroke prevention, “how far the region remains from translating what is known into what is done.”

Attention has been given to telemedicine, digital technologies, artificial intelligence, and clot-busting drugs to promote health. However, our health systems and governments fail to achieve the widespread implementation of the most fundamental and elementary approaches that promote primary prevention: prevention awareness, wide health screening for early detection, addressing social determinants of health and health inequity, and appropriate budget allocated to health-care systems. We need more healthy food promotion and accessible and affordable healthier food, and better policies addressing smoking and high alcohol intake. But we also need greater motivation and access to inviting and safe environments that promote physical activity. Unfortunately, there is no shortcut that can replace physical exercise or help us burn calories instantly after large meal portions.

Reducing modifiable risk factors of stroke requires individuals’ responsibility, but also requires addressing systemic barriers and the socioeconomic determinants of health. Governments should improve socioeconomic conditions to engage people into healthier lifestyles. Importantly, stroke, coronary heart disease, and diabetes share many modifiable risk factors. Thus, given the substantial burden of stroke and other major non-communicable diseases, governments, health-care authorities, and providers should place greater emphasis on primary prevention.

